# Hashimoto’s encephalopathy in a patient with hypothyroidism and bipolar disorder: a case report

**DOI:** 10.1192/j.eurpsy.2025.822

**Published:** 2025-08-26

**Authors:** C. Bey, T. Ach, A. Ben Abdelkarim, J. Mannaï

**Affiliations:** 1 Laboratory of Physiology and Pathophysiology of physical exercise; L.R.19ES09, University of Sousse, Faculty of Medicine of Sousse, 4000, Sousse; 2 Psychiatry department, Ibn El Jazzar University Hospital of Kairouan, Kairouan; 3 University of Sousse, Faculty of Medicine of Sousse, 4000; 4 Endocrinology department, Farhat Hached University Hospital of Sousse, Sousse, Tunisia

## Abstract

**Introduction:**

Hashimoto’s encephalopathy (HE) is a rare, steroid-responsive neuropsychiatric disorder associated with Hashimoto’s thyroiditis. The pathophysiology of HE remains unclear, but it is hypothesized to involve an autoimmune mechanism, distinct from thyroid hormone levels. The condition often presents with a variety of neurological and psychiatric symptoms, including cognitive decline, seizures, mood disorders, and movement abnormalities. Timely diagnosis and treatment are crucial to prevent further neurological impairment.

**Objectives:**

This report highlights a case of HE in a patient with bipolar disorder and hypothyroidism.

**Methods:**

A 42-year-old male patient, followed in psychiatry for bipolar disorder type I and in endocrinology for hypothyroidism secondary to Hashimoto’s thyroiditis, was admitted to the endocrinology department of Farhat Hached hospital, Sousse, due to fatigue, psychomotor retardation, and an enlarged goiter, in the context of discontinuation of his replacement therapy. Laboratory tests revealed a significantly elevated TSH level of 17.5mUI/L, indicating profound hypothyroidism. Hospitalization was therefore prompted by this endocrine decompensation to reinitiate treatment and to monitor him to prevent complications.

During the hospital stay, thyroid hormone replacement therapy was resumed. However, despite adequate treatment, the patient quickly became unstable, exhibiting vague persecutory delusions, marked irritability, changes in behavior, distractibility, attention problems, insomnia and confusion. This clinical picture raised the possibility of either a manic relapse with psychotic features, potentially triggered by the resumption of thyroid treatment, or Hashimoto’s encephalopathy.

Further investigations, including brain imaging and anti-thyroid peroxidase antibodies (ATPO) measurement, were performed. Brain imaging was normal, and ATPO were elevated. Given the clinical history and elevated thyroid antibodies, the diagnosis of Hashimoto’s encephalopathy was considered. The patient was started on corticosteroid therapy (prednisone), leading to a significant improvement in both psychiatric and cognitive symptoms within weeks.

**Results:**

This case illustrates the importance of considering HE in patients with neuropsychiatric symptoms and underlying thyroid disease. The combination of elevated ATPO levels and progressive psychiatric deterioration, with normal neuroimaging, and significant improvement with immunomodulatory treatment supports the diagnosis of HE. It is a rare condition with a reported prevalence of 2.1/100000. It presents with a wide range of neurological and psychiatric symptoms and the presentation varies among patients.

**Conclusions:**

This case underscores the need for increased awareness of HE as a differential diagnosis in patients with thyroid disorders and neuropsychiatric manifestations.

**Disclosure of Interest:**

None Declared

